# Current research and emerging directions in emotion-cognition interactions

**DOI:** 10.3389/fnint.2014.00083

**Published:** 2014-11-11

**Authors:** Florin Dolcos, Lihong Wang, Mara Mather

**Affiliations:** ^1^Psychology Department, Neuroscience Program, and Beckman Institute, University of Illinois at Urbana-ChampaignUrbana, IL, USA; ^2^Brain Imaging and Analysis Center, Duke UniversityDurham, NC, USA; ^3^Davis School of Gerontology and Department of Psychology, University of Southern CaliforniaLos Angeles, CA, USA

**Keywords:** motivated attention, emotional memory, emotional distraction, affective disorders, age-related differences, stress, amygdala/prefrontal cortex, social and personality neuroscience

Emotion is a “double-edged sword” that can either enhance or hinder various aspects of our cognition and behavior. For instance, the emotional charge of an event can increase attention to and memory for that event, whereas task-irrelevant emotional information may lead to increased distraction away from goal-relevant tasks. Sometimes, even the same emotionally arousal event can lead to opposite effects on different aspects of cognitive processing—hearing a gunshot might enhance memory for central aspects of what was happening at the time, while impairing memory for peripheral details. Stress can also lead to quite different effects depending on the context and degree of stress. For example, emotional responses associated with optimal levels of stress (eustress) may increase performance (e.g., positive emotions associated with wedding preparations), whereas emotions associated with exposure to extreme levels of stress impair performance (e.g., overwhelming worry in the anticipation of a difficult exam). Importantly, these effects are also susceptible to cognitive influences, typically exerted in the form of emotion control, which may affect both the immediate and the long-term impact of emotion on cognition. Although during the last decades important progress has been made in understanding emotion-cognition interactions, a number of aspects remain unclear.

The present e-book and research topic comprise a collection of manuscripts discussing emerging evidence regarding the mechanisms underlying emotion-cognition interactions in healthy functioning and alterations associated with clinical conditions, in which such interactions are dysfunctional. Co-hosted by the *Frontiers in Neuroscience—Integrative Neuroscience* and *Frontiers in Psychology—Emotion Science*, our special research topic attracted a large number of outstanding contributions, based on approaches spanning from behavioral and lesion to pharmacological and brain imaging, and including empirical, theoretical, and review papers alike. The contributions are grouped around the following seven main themes distributed across the two hosting journals: *(1) Emotion and Selectivity in Attention and Memory, (2) The Impact of Emotional Distraction; Linking Enhancing and Impairing Effects of Emotion, (3) What Really is the Role of the Amygdala?, (4) Age Differences in Emotion Processing; The Role of Emotional Valence, (5) Affective Face Processing, Social Cognition, and Personality Neuroscience, (6) Stress, Mood, Emotion, and the Prefrontal Cortex; The Role of Control in the Stress Response, (7) Emotion-Cognition Interactions in Clinical Conditions*. This comprehensive approach allowed an integrative understanding of the available evidence and identification of concrete venues for future investigations.

## Emotion and selectivity in attention and memory

Perhaps the most studied issue in the history of emotion-cognition interactions is how emotion influences the scope or selectivity of attention and memory. Does arousal or negative emotion lead to the narrowing of the scope of attention? Do arousing stimuli create a trade-off in memory such that they are remembered better than similar neutral stimuli, while they impair memory for surrounding information? How do goals and the type of emotion relate to these selectivity effects? The papers in this section provide an excellent overview of current issues and controversies regarding this topic.

Even in the absence of strong emotion, different stimuli compete for representation in the brain (Desimone and Duncan, 1995; Beck and Kastner, 2009). Attention helps modulate this competition, even at the synaptic level (Briggs et al., 2013). Arousal interacts with these other factors. When a stimulus is inherently emotionally arousing, it has a competitive advantage over stimuli nearby in space or time (Bradley et al., 2012). For instance, people tend to remember foreground objects better when they are emotional than neutral, but at the cost of poorer memory for the background scenes (Steinmetz and Kensinger, 2013). These effects favoring emotionally salient objects over the surrounding less salient context become stronger with a night of sleep (Payne et al., 2012). Such trade-off effects also occur in people with post-traumatic stress disorder (PTSD) but not in those who experienced trauma in the past without developing PTSD (Steinmetz et al., 2012). These intriguing findings suggest that people in whom arousing items do not have as much of a competitive advantage might have a lower propensity toward PTSD.

The competitive advantages for emotional stimuli work across time, as well as space. Wang et al. (2012) review the phenomenon known as emotion-induced blindness, in which emotionally arousing stimuli can disrupt perception of other target stimuli that appear soon afterwards in the same location. They argue that emotion-induced blindness results from competition between the targets and the emotional distractors, with the distractors gaining priority due to their goal relevance. But this raises the question of whether emotional stimuli really do get more attention than would be predicted simply by their visual salience. To test this, Niu et al. (2012) had participants code which regions within pictures were most affectively salient and then had automated software compute which regions were most perceptually salient and measured eye movements to assess attention. They found that affectively salient regions attracted more attention than perceptually salient regions, especially among pictures rated high in arousal. But findings that recalling autobiographical memories in response to emotional cue words impairs subsequent perception more than recalling in response to neutral cue words indicate that emotion can impair perception even if there are no external differences in perceptual salience (Young et al., 2012).

Thus, papers in this section continue a long line of research indicating that emotionally arousing stimuli attract more attention and are remembered better than competing stimuli. However, the question of whether this has to do with arousal, *per se*, and not just with the nature of the stimuli, requires methods in which arousal is manipulated independently of the stimuli characteristics. To do this, Echterhoff and Wolf (2012) manipulated stress after participants watched a video of a burglary, as well as whether participants anticipated something distressing during the video. They found that the recognition advantage for central event items over peripheral event items was largest when distress was anticipated and there was post-event stress. Thus, arousal both during an event and immediately afterwards can enhance the relative memory advantage for salient events that just occurred.

The notion that arousal enhances processing of salient stimuli and impair processing of other stimuli is consistent with Mather and Sutherland's (2011) arousal-biased competition theory, in which arousal increases the stakes of neural competition between stimuli representations, such that there are both “winner-take-more” and “loser-take-less” effects. In their present study, Lee et al. (2012) tested arousal-biased competition mechanisms by manipulating arousal during a 2-session perceptual learning task. One session included interspersed emotionally arousing negative pictures whereas the other session included interspersed neutral pictures. Participants' task was to find the discrepant line from among an array of lines. In one condition, the discrepant line was always tilted 55° among a field of 50° distractors. In another condition, the discrepant line was always tilted 55° among a field of 80° distractors. In both conditions, participants had to identify the exact tilt of the target line from a set of five alternatives every so often. Thus, in one condition, participants were learning about a salient item (a line very different from its competitors) whereas in the other they were learning about a non-salient line. Competition models of salience (e.g., Itti and Koch, 2000) predict that competition will favor the salient line, enhancing its representation, but that mutual suppression will impair the non-salient line, leading its representation to be impaired. Indeed, in the salient-target condition, the interspersed emotional pictures enhanced participants' perceptual tuning curves as indicated by more accurate memory for the tilt of the target line. In contrast, in the non-salient-target condition, emotion impaired learning about the target line, leading to broader estimated tuning curves. Such work follows up on other recent findings that emotional arousal enhances processing of neutral but perceptually salient stimuli while impairing processing of competing less salient stimuli (Sutherland and Mather, 2012; Lee et al., 2014). Also, recent findings indicate that emotional pictures (positive or negative) strengthen high priority memory traces but weaken low priority memory traces, when priority was determined by goal relevance (Sakaki et al., 2014). *Thus, arousal can have opposite effects on low and high priority representations, with both bottom-up salience and top-down goal relevance as key determinants of priority*.

However, two reviews in this book present different views on how emotion influences selectivity. In their review, Harmon-Jones et al. (2012) argue that what really matters is the motivational intensity of emotion. Affective states high in motivational intensity should narrow cognitive scope while those low in motivational intensity should broaden it. Kaplan et al. (2012) also highlight the role of motivation but argue that a critical factor is whether goals have been achieved yet or not. They posit that emotions experienced before goal attainment or loss should lead to memory narrowing while emotions experienced afterwards should broaden the scope of attention and encoding. One key issue in trying to understand whether these motivational accounts contradict or are complementary to arousal-biased competition account is that the measures differ. Most of the studies examining how motivational intensity influences attention have assessed how much people attend to the local or global features of the same information. For instance, in the Navon (1977) letters task participants see large letters composed of smaller letters and attentional biases to the large or small letters is measured. Motivational intensity shifts these local/global biases. But the arousal-biased competition account requires knowledge about the relative baseline priority of the small and large letters. Given that there are individual differences in whether the large or small letters are noticed more quickly, there is no clear high priority item across participants. Arousal-biased competition processes should favor the local features even more for those who already tend to favor the local letters and vice versa for those who tend to favor the global letters. Likewise, it is not clear what motivational intensity would predict in the type of paradigm used by Sutherland and Mather (2012), in which some letters are more perceptually salient because they contrast more with the background, but they are all equally central in the image and all have the same size.

Finally, the opinion paper by Chiu et al. (2013) proposes that other factors should also be considered when investigating the impact of emotion on memory. Namely, while extant theories tend to focus on how attentional biases in the perception of emotional stimuli influence memory, future research would benefit from differentiating between source, contextual, and relational information, as a way to further our understanding of the effects of emotion on various types of memory phenomena (Chiu et al., 2013).

## The impact of emotional distraction; linking enhancing and impairing effects of emotion

A related key question in the emotion literature is how emotionally arousing stimuli can also cause distraction and disrupt performance. Novel evidence emerging from human (Hart et al., 2012; Jasinska et al., 2012a,b; Shafer and Dolcos, 2012; Dolcos et al., 2013) and animal (Matthews et al., 2012) research confirm and advance the initial view (Dolcos and McCarthy, 2006) that processing of task-irrelevant emotional information is associated with opposing patterns of response in ventral emotion processing regions, such as the amygdala (showing enhanced activity), and in dorsal executive brain regions, such as the dorsolateral prefrontal cortex (dlPFC, showing reduced activity) (reviewed in Iordan et al., 2013). New evidence shows that the response to emotional distraction can be modulated by actual or expected task difficulty (Hart et al., 2012; Jasinska et al., 2012b; Shafer and Dolcos, 2012), stress hormones (Henckens et al., 2012), and genetic differences (Jasinska et al., 2012a), as well as by personality differences in traits indexing increased sensitivity to processing task-irrelevant information, such as attentional impulsivity (Dolcos et al., 2013). Interestingly, there is also evidence that emotional “distraction” may not always impair performance on the primary task (Jackson et al., 2012; Truebutschek and Egner, 2012). Finally, there is also evidence that increased emotional reactivity in clinical patients with emotional and attentional disorders is associated with increased impact of emotional distraction (Singhal et al., 2012).

*Notably, studies in this section also provide initial evidence regarding the neural mechanisms linking enhancing and impairing effects of emotional distraction*. Whereas emotional distraction initially *impair*s perception (Shafer and Dolcos, 2012) and working memory (Dolcos et al., 2013), it *enhances* long-term episodic memory for the distractors themselves. The emotional charge of the distracting information and the difficulty of the main cognitive task influence both the initial emotional distraction (Shafer et al., 2012) and long-term memory (Shafer and Dolcos, 2012). Also, Dolcos et al. (2013) showed that the same amygdala area was involved in both enhancing and impairing effects of emotion, but its engagement was associated with opposing influences on activity in brain regions responsible for maintaining performance in the initial main cognitive task (dlPFC, showing reduced response to emotional distraction) vs. those underlying the long-term enhancing effects of emotion (MTL memory system, showing enhanced response) (see also Dolcos and Denkova, 2014).

Interestingly, the study by Dolcos et al. (2013) also shows that the involvement of cognitive control mechanisms to cope with emotional distraction (reflected in increased activity in the ventrolateral prefrontal cortex, vlPFC) not only protects against the emotional distraction but also leads to enhanced memory for the distractors themselves. This finding provides novel evidence regarding the neural mechanisms underlying a behavioral phenomenon that was observed in early studies investigating the initial and long-term effects of emotion regulation. Namely, the engagement of specific emotion regulation strategies to cope with the initial exposure to emotional situations may also lead to increased memory for the emotional aspects of that experience (Dillon et al., 2007). This issue also has relevance for understanding clinical conditions, such as PTSD, where dysfunctional links between immediate and long-term effects of emotion may be associated with erroneous initial encoding of memories for traumatic events due to heightened arousal, as discussed in the opinion article by Dolcos (2013).

## What really is the role of the amygdala?

Current theories give the amygdala a fundamental role in enhancing attention and memory of emotional stimuli (Murty et al., 2010; Dolcos et al., 2011, 2012; Mather and Sutherland, 2011; Pourtois et al., 2013). Yet, there are still multiple debates about the exact nature of its role. For instance, there has been a long-standing debate about the automaticity of emotion processing and about the nature and significance of dissociating between subcortical automatic and non-conscious route to the amygdala from cortical routes for processing emotional stimuli (for recent reviews, see Pessoa and Adolphs, 2010; Tamietto and de Gelder, 2010). Consistent with the traditional view regarding emotion-attention interaction (Ohman et al., 2001), in their present contribution, De Gelder et al. (2012) argue that work using moving bodies expressing emotion or not indicates that there is an early emotion processing route that is independent of attention and involves a subcortical route to the amygdala; for recent evidence reconciling the traditional and competing views, see Shafer et al. (2012).

In their review, Chau and Galvez (2012) argue that while the amygdala is widely acknowledged to play a key role in the initial acquisition and consolidation of fear-related memories, it also is involved in non-fear-related memories, such as eyeblink conditioning. They concur with previous accounts that the amygdala works as a salience detector to enhance learning of biologically significant or behaviorally relevant events (Davis and Whalen, 2001; Sander et al., 2003; Pessoa and Adolphs, 2010; Pourtois et al., 2013), so that other brain regions are more likely to consolidate those important learned responses.

One puzzle in the literature is the finding that patients with Alzheimer's disease, still show emotional enhancement effects in memory (e.g., Kazui et al., 2000; Nashiro and Mather, 2011). In their review, Klein-Koerkamp et al. (2012) argue that Alzheimer's patients may have compensatory or “reinforced” neural projections between brain areas involved in the elicitation of emotional enhancement. This is an intriguing hypothesis, but it would also be useful to consider other aspects of arousal that may contribute to enhanced memory, beyond the amygdala. In their paper, Gold and Korol (2012) review neuroendocrine influences on emotional memory. They argue that increases in blood glucose levels that result from epinephrine release during arousal augment neurotransmitter release and energy metabolism in brain regions involved in learning and memory. However, the impact of glucose on memory is impaired in normal aging and even further in Alzheimer's disease (Gold and Korol, 2012), thus there is no obvious answer as to how memory continues to be enhanced by emotion in those with Alzheimer's disease, albeit to a less robust degree than for healthy older adults.

The amygdala does not always lead to enhanced processing. As discussed in the previous section, the same amygdala area appears to mediate both impairing and enhancing effects of emotional distraction, but through different effects on activity in the dlPFC (decreased) vs. MTL (increased) (Dolcos et al., 2013). In addition, while it enhances new emotional learning, lesion work with animals indicates that the amygdala impairs the flexibility of learning, such that reversal learning or extinction are facilitated after amygdala lesions (Izquierdo and Murray, 2005; Stalnaker et al., 2007). When intact, the amygdala seems to work against orbitofrontal mechanisms that help update memories. This model is consistent with recent findings that updating the context of emotionally arousing information is more difficult and involves greater activation of frontopolar/orbitofrontal cortex than updating neutral information (Mather and Knight, 2008; Novak and Mather, 2009; Sakaki et al., 2011; Nashiro et al., 2012). As shown in the present contribution by Nashiro et al. (2013b), the behavioral disadvantage in updating emotional compared to neutral associative information is maintained in older adults, as is the increased activity in the orbitofrontal cortex and increased negative amygdala-PFC functional connectivity when doing emotional reversal learning, compared to neutral reversal learning (Nashiro et al., 2013a).

*Thus, across the life span, the amygdala works to maintain associations within existing emotional memories, such as what a cue predicts, where a particular picture was, or which item something was paired with, making emotional memories more resistant to flexible updating*. Also relevant are the present findings that it is more difficult to learn whether items previously associated with high rewards than those associated with low rewards were in a new list (Madan et al., 2012). Thus, both reward and arousal make it more difficult to learn new contextual information about items. However, more research is needed to understand if the mechanisms of impaired updating are the same for reward and arousal.

## Age differences in emotion processing; the role of emotional valence

People's intuition tends to be that emotion should follow the same downward trajectory with age as cognition, such that negative emotion increases as people grow older. However, longitudinal studies reveal that, in fact, rates of negative affect (NA) tend to decrease through much of adulthood (Carstensen et al., 2000; Charles et al., 2001). These shifts in emotional experience are reflected in cognitive processes as well, leading to age-by-valence interactions (known as the “positivity effect”) in attention and memory (Mather and Carstensen, 2005). Specifically, older adults tend to favor positive stimuli more and negative stimuli less than younger adults, in their attention and memory. Since initial findings of an age-related positivity effect in attention and memory were published about a decade ago (Charles et al., 2003; Mather and Carstensen, 2003), there has been a great deal of interest in the effect. In their present review paper, Reed and Carstensen (2012), discuss how the pattern of findings indicate that the positivity effect is not a manifestation of cognitive decline, but instead requires controlled processing (Mather and Carstensen, 2005; St. Jacques et al., 2010). Given its reliance on goal-directed processes, the positivity effect is malleable and context sensitive. For instance, manipulating concurrent cognitive load can diminish or reverse it (Mather and Knight, 2005; Knight et al., 2007), something that cannot be accounted for by a cognitive decline account. Reed and Carstensen (2012) also point out that *more work is needed to test whether positivity in cognitive processing helps emotional well-being and influences decisions*.

Several of the empirical papers in this collection addressed other relevant issues. Noh et al. (2012) investigated whether the links between positive and NA and attentional functioning varies as a function of age. They found that higher levels of positive and lower levels of NA were associated with enhanced orienting efficiency in older adults, but neither of them had any influence on executive attention. Overall, the findings by Noh et al. (2012) suggest that positive and NA may influence attentional functioning in distinct ways, but that these patterns may depend on the age group. Pollock et al. (2012) examined adult age differences in processing emotional faces using a psychological refractory period paradigm. Younger adults showed significantly higher P1 responses for angry than for happy faces, whereas older adults showed no difference, revealing another measure on which positivity effect age-by-valence interactions turn up; P1 is a positive event-related potential component occurring ~100 ms post-stimulus onset, which was linked with amygdala modulation of attention to emotional faces (Rotshtein et al., 2010). Kalpouzos et al. (2012) examined what they call the “negativity effect” or the memory advantage for negative emotional information over neutral information. They assessed recognition memory for neutral and negative scenes after 1 and 3 weeks in younger and older adults using fMRI. As they did not include positive stimuli, they were unable to assess whether age-by-valence interactions changed over multiple testing sessions. However, they found that older participants were better able to discriminate old from new scenes if they were neutral than negative, whereas this was not the case for younger participants. Whereas younger adults showed changes in brain activity during retrieval across the different retention intervals, the older adults did not. While, overall, the effects of delay were similar across age groups, the older adults were close to floor in their performance by the last test, which might have reduced the ability to see effects.

Although the positivity effect occurs in many studies examining attention and memory, it is not clear whether similar factors influence the ability to read facial emotions. Older adults show impaired recognition of emotions such as sadness and anger, but they are equally good or better than younger adults at recognizing disgust (Ruffman et al., 2008). In their present contribution, Ebner et al. (2012) provided new evidence that brain activation patterns are similar across younger and older adults when identifying facial expressions, indicating that, on the whole, similar PFC mechanisms are involved for both age groups. Moreover, Biss et al. (2012) provided new evidence of an emotion-cognition interaction that operates similarly across age groups. Their study revealed that, for older adults, positive mood states led to greater implicit memory for distracting words shown during a task, an effect previously shown in younger adults. Thus, positive affect seems to influence attention regulation similarly across age groups.

## Affective face processing, social cognition, and personality neuroscience

The emerging fields of social and personality neurosciences also provide important evidence regarding emotion-cognition interactions, while furthering our understanding of the neural mechanisms of adaptive social behaviors and of individual variation in the susceptibility or resilience to emotional disturbances. Human faces are essential in conveying important social information, and facial stimuli have been used to investigate a variety of aspects of social cognition, from simple emotional reactions (Morris et al., 1996) to more complex affective states (Bartels and Zeki, 2004; Nitschke et al., 2004). However, despite a long history of investigation of basic facial emotional expressions (Ekman and Oster, 1979), only relatively recently research has begun to consider other socially relevant dimensions conveyed by faces, such as trustworthiness (Todorov et al., 2009).

Processing of emotional (particularly fearful) faces involves the amygdala and fast, possibly automatic, mechanisms (Whalen et al., 1998; Sergerie et al., 2008; Adolphs, 2010; Iordan et al., 2013); but see Pessoa (2013). However, less is known about the immediate motor and cognitive consequences of such fast processing. New evidence from the present collection (Bocanegra and Zeelenberg, 2012) shows that masked fearful faces enhance the activation and inhibition phases of motor response to a target, even before conscious perception of the target. Evidence from another study suggests that when identification of facial expression occurs, cognitive control mechanisms also come into play (Ebner et al., 2012). Specifically, the authors argue that increased engagement of the dorsomedial PFC (dmPFC) for angry faces reflects cognitive control mechanisms involved to regulate negative emotions. Interestingly, similar findings were observed in both young and older participants, although older participants tended to show more extended activations of dmPFC to angry faces, possibly reflecting increased emotion control (St. Jacques et al., 2010).

Evaluation of emotional faces in social contexts and their encoding into memory are needed in order to navigate in social environments, and to decide whom to thrust and approach or to avoid (Vrticka et al., 2009). Approach and avoidance behaviors are of particular importance in social cognition and have been linked to social inclusion or exclusion, as discussed in the present review by Powers and Heatherton (Powers and Heatherton, 2012). The authors suggest that approach and avoidance behaviors could occur also concomitantly—e.g., people feeling social exclusion may simultaneously employ both affiliating and defending strategies in order to establish new social connections and to avoid negative consequences of social exclusion. Regarding social exclusion, the present review by Cacioppo and Cacioppo (2012) also emphasizes that the impact of subjectively perceived isolation is much greater than that of real isolation, and that perceived (or experimentally induced) social isolation modulates similar brain mechanisms to those involved in the perception of emotional and social stimuli (dmPFC, temporoparietal junction—TPJ, ventral striatum) (Cacioppo and Cacioppo, 2012). Moreover, Masuda et al. (2012) provide evidence regarding the role of cultural differences in processing social cues, and highlights the importance of considering contextual differences in interpreting facial signals as a function of cultural variations. Finally, by considering three affective signals conveyed by faces (emotional expressions, attractiveness, and trustworthiness), Tsukiura (2012) proposes a model suggesting mutual interactions between affective and memory systems, which explain why emotional and socially relevant faces are better remembered than neutral ones.

A long standing view is that approach and avoidant temperamental/motivational tendencies are associated with patterns of lateralization in the frontal regions, with the left side being associated with approach and the right side with avoidance (Harmon-Jones et al., 2010). However, as discussed in the present review by Miller et al. (2013), recent studies provide evidence that different sub-regions within the PFC have different roles in various aspects of emotion processing, and therefore can show different lateralization patterns, which may go beyond the simple left-right frontal conceptualization initially proposed. Other authors adopt a goal-related perspective regarding motivational tendencies, and investigate behavior in terms of *promotion/*accomplishment-oriented tendencies (planning to “make good things happen”) vs. *prevention*-oriented tendencies (planning to “avoid bad things from happening”) dichotomy (Higgins, 2012). In their present investigation, Strauman et al. (2013) provide evidence regarding the neural mechanisms underlying activation of promotion and prevention goals by priming participants' own *ideal* and *ought* goals, which are mapped onto dissociable neural substrates in healthy functioning (Eddington et al., 2007) and are altered in clinical cohorts (Eddington et al., 2009). *Overall, the evidence emerging from these contributions underscore the importance of considering the interplay between emotion and cognition at various levels, from basic processing of social cues to their integration in larger behavioral and cultural contexts*. Such integration not only influences individuals' own adaptive social behavior but may also have consequences on their moral social decisions, as also discussed here by Cummins and Cummins (2012).

## Stress, mood, emotion, and the prefrontal cortex; the role of control in the stress response

Another important question in the emotion literature is how long–term affective states, such as those produced by stressful experiences, mood manipulations, or associated with personality traits, influence cognition and behavior. It is widely accepted that optimal levels of stress benefit cognitive functioning, whereas extreme and/or chronic levels of stress impair it. But how much stress is too much stress, and how do specific stressors and individual differences influence the experienced stress and its impact on cognitive functioning?

Novel evidence from the present collection points to both beneficial and detrimental effects of stress and mood on cognitive functioning and the associated neural mechanisms, which are influenced by manipulations of stress hormones (Henckens et al., 2012), personality traits indexing emotion regulation and NA (Crocker et al., 2012; Wang et al., 2013), and previous history of stress (Wang et al., 2013), as well as by age (Biss et al., 2012) and genetic (Qin et al., 2012) differences. Henckens et al. (2012) provide evidence for (i) rapid effects of cortisol reflected in impaired selective attention and increased emotional interference, driven by bottom-up processing in the amygdala and its interaction with the PFC, and (ii) slow effects of cortisol promoting sustained attention, by reducing bottom-up driven processing in visual brain areas and potentially leading to restoration of brain functioning after stress. The authors propose a more adaptive view of the impact of cortisol on attention and emotion, with an initial effect optimizing detection of potential threat at the cost of impaired cognitive processing, and a delayed effect normalizing cognitive brain functions following stress (Dolcos, 2014). Crocker et al. (2012) points to joint and dissociable effects of state and trait NA on attention. Specifically, when combined, state and trait NA produced the strongest impact on performance in an attentional task, but they were linked to different aspects of processing—i.e., state NA was linked to excessive stimulus-driven processing of salient irrelevant information, whereas trait NA was linked to difficulty in engaging top-down attentional control to deal with irrelevant information. Wang et al. (2013) show that the impact of early-life stress on activity in the vmPFC (a region involved in emotion control) is linked to variations in personality traits indexing diminished (rumination) or enhanced (mindfulness) emotion control. This, in turn, influences susceptibility or resilience to emotional challenges, in response to exposure to repeated mild stressful situations, linked to reduced vs. enhanced vmPFC activity, respectively. Finally, Qin et al. (2012) further show that the effect of moderate stress on working memory performance and PFC functioning is modulated by genetic variations in the genes encoding the Catechol-O-methyltransferase (COMT; an enzyme that degrades catecholamines such as dopamine, epinephrine, and norepinephrine), with some allele combinations being associated with positive whereas others with negative effects.

Besides individual variations (Kanske, 2012; Qin et al., 2012; Wang et al., 2013), other factors linked to available resources, task-relevance, and top-down modulation can also explain differential impact of affect and stress on cognition, as discussed in the review by Cohen and Henik (2012). Moreover, Froeliger et al. (2012) provides preliminary evidence that the engagement in meditation practice could help avoid the negative impact of emotion on cognition, which is consistent with the idea that the engagement of reactive or proactive strategies of cognitive control can alleviate detrimental effects of emotion (Patterson et al., 2012).

*An interesting idea that also emerges from the present studies investigating these issues is that the effect of stress on cognitive functioning and the associated neural mechanisms can be modulated by the feeling of control and/or the subjective experience of stress (Buetti and Lleras, 2012; Henderson et al., 2012; Kerr et al., 2012)*. Specifically, the presence of controllability can help improve cognitive performance, when stress is perceived as moderate (Henderson et al., 2012), and abolish distortions in time perception of negative compared to positive events (Buetti and Lleras, 2012). Moreover, the presence of controllability also affects the PFC functioning, with the vmPFC showing increased activity during controllable anticipation response (Kerr et al., 2012). This finding can be linked to Wang et al.'s finding revealing decreased vmPFC response in people with early-life stress and high trait rumination (Wang et al., 2013). Interestingly, while there is evidence that extreme subjective experience of stress can have detrimental effects on cognitive functioning, especially in uncontrollable situations (Henderson et al., 2012), the low perceived stress in acute stressful situations can also have detrimental effects on decision making, by favoring risky decisions (Lempert et al., 2012), which has relevance for understanding addictive gambling behavior.

## Emotion-cognition interactions in clinical conditions

Although the terms emotion and cognition describe concepts that involve overlapping functions and recruit overlapping circuits within the brain, thinking about their distinctive features and how they interact can be quite fruitful. This is especially the case in the domain of psychiatric disorders, where dysfunctional emotion-cognition interactions are among the most obvious impairments. For instance, Dolcos (2013) points out that in PTSD both the enhancing and impairing aspects of emotion-cognition interactions are exacerbated and detrimental. This statement is also true for the majority of other psychiatric disorders. A series of original and review articles in the present collection discuss the relationship between emotion and cognition in a variety of psychiatric disorders including, depression, PTSD, schizophrenia, autism, Alzheimer's disease, and substance abuse.

### Major depressive disorder

Major depressive disorder (MDD) has been posited to result from dis-coordination between cognitive and affective neural systems (Mayberg, 1997), which could be a cause and/or a result of negative affective biases, the core cognitive deficits of MDD. In this book, Foland-Ross and Gotlib (2012) summarize evidence from the literature regarding the negative bias in MDD and note that depression is characterized by difficulty in disengaging from negative material, once it initially captures attention, rather than from rapid orientation to negative stimuli. However, this phenomenon is not specific to depression, as also shown by the Singhal and colleagues' study using event-related potential recordings in adolescents with comorbid psychiatric disorders, such as attention-deficit/hyperactivity disorder (ADHD), anxiety, or other psychiatric disorders (Singhal et al., 2012). This study provided evidence that effortful activity is required in these patients to redirect attention from task-irrelevant negative stimuli to processing goal-relevant information (Singhal et al., 2012). The negative affective bias has also been extended to emotionally positive materials (LeMoult et al., 2012). The study by LeMoult et al. (2012) shows that when happy emotional expressions are used as primes, healthy adults show the strongest interference, whereas the MDD group fail to show such positive bias.

### PTSD

Like depression, negative affective biases can also be found in PTSD (Aupperle et al., 2012), and reflects difficulty in disengaging from, rather than facilitated detection of, negative stimuli. In PTSD, however, there is also heightened trauma-related reactivity, along with paradoxical changes in memory processing (Cohen et al., 2006; Parsons and Ressler, 2013). On the one hand, emotionally salient traumatic memories tend to induce uncontrolled recollection and re-experiencing of memories for traumatic events. On the other hand, PTSD patients tend to avoid stimuli associated with the trauma, which may induce amnesia for details of the traumatic event (Brewin et al., 2011). All three review articles on PTSD (Brown and Morey, 2012; Hayes et al., 2012; Dolcos, 2013) in this book point to exacerbated enhancing and hindering effects of emotion on cognition in PTSD. Uncontrolled recollection of distressing memories may lead to impairment in cognitive task performance due to enhanced emotional distraction (Dolcos, 2013). Furthermore, emotional interference in PTSD can be extended beyond trauma-related materials to trauma-unrelated emotional materials, as emphasized in the review by Brown and Morey (2012), who propose two disrupted networks in PTSD, a trauma-activated network and an emotion-activated network. There is also evidence that PTSD patients may be more prone to falsely remembering novel information (Hayes et al., 2012). However, in the study from the present collection mentioned above (Steinmetz et al., 2012), PTSD patients did not show greater emotional trade-offs (greater memory to emotional items than neutral background) than controls or trauma-exposed people without PTSD, unlike the trauma-exposed non-PTSD group which showed such trade-off. More studies are needed to elucidate these issues, and understanding the neural mechanisms underlying emotional and cognitive deficits in PTSD may provide insight for developing novel cognitive-based behavioral therapies.

### Schizophrenia

According to Anticevic and Corlett (2012), patients with schizophrenia have an intact ability to perceive emotion “in the moment.” The core deficit of the illness is a dysfunction in “*cold*” cognitive processes, predominantly in working memory, which is associated with markedly reduced activity in the dlPFC in these patients. Thus, it has been suggested that it is the dysfunction of working memory that gives rise to difficulty in maintaining affective/reward-related information representation in guiding motivational behaviors. Future studies could demonstrate the link between the lack of “maintenance” signals following affective/reward cues to deficits in future goal-directed behavior and negative symptoms (such as anhedonia) in schizophrenia. Unlike depressed patients who are distracted by negative information and unable to disengage from negative events to on-going goal-guided events, schizophrenic patients have difficulty in filtering task-irrelevant distracting information. They tend to abnormally assign neutral stimuli as salient and have deficits in generating appropriate prediction error signals, so that they blur the distinctiveness of responses to events that violate vs. confirm expectations, which could lead to delusions.

### Autism spectrum disorders (ASD)

These neurodevelopmental disorders affect neural systems largely related to social-emotional and social cognition processes. While the field predominantly views ASD as involving deficits primarily in the “social cognition network,” Gaigg's present paper (2012) questions this notion. In his review, Gaigg argues that the social deficits in ASD could be the consequence of abnormal emotional responses to the environment “which modulate a wide range of cognitive processes including those that are relevant to navigating the social world.” Gaigg concludes that ASD involves relatively preserved function in basic emotional evaluation (i.e., arousal-related responses response to emotionally salient pictures and words), but is associated with difficulties in more complex emotional processes related to social environment, including identification and interpretations of emotions in others. Individuals with ASD have difficulties in the response to ambiguous and unpredictable events in the environment, and in learning about the emotional significance of stimuli that predict biologically relevant events. Gaigg posits an intriguing hypothesis that the basic amygdala functions that are mediated by sub-cortical networks involving sensory afferents from the thalamus and efferents to brain-stem nuclei are preserved in ASD, but the connections with cortical or sub-cortical regions modulating amygdala appear to be compromised. However, this idea needs confirmation from future studies.

### Alzheimer's disease (AD)

Turning to alterations in emotion-cognition interactions in clinical conditions affecting later developmental stages, an intriguing aspect concerns the possibility that relatively better preserved emotion processing could buffer memory deficits in AD patients. According to the present review by Klein-Koerkamp et al. (2012), both enhancing and impairing effects of emotion on memory can be found in AD, and that these discrepant findings could be due to differences among studies in a number of factors, as follows. (1) Task difficulty (easy vs. hard): tasks that are too easy for healthy controls or too difficult for AD patients could lead to ceiling vs. floor effect respectively, which may obscure the memory-enhancing effect of emotion. (2) Task type (recollection vs. recognition): typically, the memory-enhancing effect of emotion tends to be stronger in recollection than in recognition tasks, and hence this may also explain discrepant findings observed in AD, depending on the tasks involved. (3) Stimulus properties (exposure time and self-relevance): emotionally intense and self-related stimuli are more likely to induce emotional enhancement effects on declarative memory. Overall, Klein-Koerkamp et al. (2012) emphasize that understanding the underlying mechanisms and the factors influencing the memory-enhancing effect of emotion on memory in AD can potentially guide the development of new therapies, based on using emotionally salient cues to enhance/guide memory in these patients.

### Substance dependence

The emotional disturbances commonly seen across all drug dependencies are: (1) heightened reward sensitivity to drug-related stimuli (incentive sensitization), (2) reduced sensitivity to natural reward stimuli, and (3) weakened cognitive control, reflected in diminished PFC engagement and heightened sensitivity in the brain's stress systems that respond to threats (Murphy et al., 2012; Volkow et al., 2013). In their contribution to the present collection, Murphy et al. (2012) argue that the baseline dopamine levels in these patients are low, and when drug cues (but not natural reward stimuli) are presented, high levels of dopamine are released, which results in drug craving. This suggests an overall reward deficiency (reduced sensitive to natural reward stimuli) but sensitized reward systems to drug cues in substance dependence. Murphy et al. (2012) also propose that emotional dysregulation could be the causal factors for impulsive behavior and poor decision–making in these patients.

As we have reviewed how disturbances of emotion-cognition interactions result in various types of disorders, we cannot neglect the significant role of puberty in the maturation of emotion-cognition interactions (Ladouceur, 2012). One factor during puberty could be related to changes in the production of sex hormones, which can affect the response to emotionally salient distractors, possibly because of the influence of sex hormones on the functional connectivity of PFC regions. Another important factor that affects the maturity of the affective and cognitive systems is early-life stress. It has been well-documented that early-life stress is associated with psychiatric disorders in adults. Here, Flynn and Rudolph (2012) provide new evidence that maternal depression (but not anxiety) predicts increased negative bias in adolescents with high levels of interpersonal emotional reactivity. However, maternal depression does not predict cognitive biases in those exhibiting low levels of interpersonal emotional reactivity during adolescence. This finding that maternal depression but not maternal anxiety predicts negative bias needs replication to elucidate the mechanisms of possible “depression transmission.” Examination of the impact of sex hormones, genetic changes, personality, and of early-life stress on the development and maturity of emotional and cognitive systems are all active research areas, which may shed light on the neural mechanisms and potential treatment for psychiatric disorders, which tend to onset during early developmental stages.

One of the critical issues hampering clinical diagnosis for psychiatric disorders using objective measures (biomarkers) is the heterogeneous manifestations of the mental disorders. There is a need for new models to better accommodate large individual variance. In his review article, Northoff (2012) proposes an interesting theory that our awareness of emotional feelings is determined by the body-environment relation, which may be translated through neural activity. Although there are considerable variations in disruptions of psychological processes among the clinical conditions discussed above, to some degree all these disorders have dysfunctions in the prefrontal-striato-limbic-thalamus networks. Hence, Northoff (2012) proposes that it is necessary to study these disorders together under a new comprehensive model, such as the frame of body-environment relationship. The proposed framework has important empirical and conceptual implications, and ideally future approaches will also involve systematic studies linking abnormal findings at molecular/cellular, system, and psychological levels, to better understand the neuropathology of different psychiatric disorders (Anticevic and Corlett, 2012).

Overall, as illustrated by the present collection of contributions, emotion-cognition interactions can be identified at different levels of processing, from perception and attention to long-term memory, decision making processes, and social cognition and behavior. Notably, these effects are subject to individual differences that may affect the way we perceive, experience, and remember emotional experiences, or cope with emotionally challenging situations. Moreover, these opposing effects tend to co-occur in affective disorders, such as depression and PTSD, where uncontrolled recollection of and rumination on distressing memories also lead to impaired cognition due to emotional distraction. Understanding the nature and neural mechanisms of these effects is critical, as their exacerbation and co-occurrence in clinical conditions lead to devastating effects and debilitation. Hence, bringing together such diverse contributions has allowed not only an integrative understanding of the current extant evidence but also identification of emerging directions and concrete venues for future investigations.

### Conflict of interest statement

The authors declare that the research was conducted in the absence of any commercial or financial relationships that could be construed as a potential conflict of interest.
